# The feedback-related negativity reflects “more or less” prediction error in appetitive and aversive conditions

**DOI:** 10.3389/fnins.2014.00108

**Published:** 2014-05-20

**Authors:** Yi Huang, Rongjun Yu

**Affiliations:** School of Psychology and Center for Studies of Psychological Application, South China Normal UniversityGuangzhou, China

**Keywords:** feedback-related negative, appetitive, aversive, reinforcement learning, ERP

## Abstract

Humans make predictions and use feedback to update their subsequent predictions. The feedback-related negativity (FRN) has been found to be sensitive to negative feedback as well as negative prediction error, such that the FRN is larger for outcomes that are worse than expected. The present study examined prediction errors in both appetitive and aversive conditions. We found that the FRN was more negative for reward omission vs. wins and for loss omission vs. losses, suggesting that the FRN might classify outcomes in a “more-or-less than expected” fashion rather than in the “better-or-worse than expected” dimension. Our findings challenge the previous notion that the FRN only encodes negative feedback and “worse than expected” negative prediction error.

## Introduction

Human beings can learn from the consequences of their actions. Rapidly evaluating the external feedback information and using it to guide future actions are important for human behavior. Such learning depends on the ability of the brain to discriminate between positive feedback indicating that the behavior was appropriate and negative feedback indicating that the behavior was inappropriate (Nieuwenhuis et al., 2004). Recent studies have used event-related potentials (ERP) to examine how feedback is processed in the human brain. A component of event-related potential, named feedback-related negativity (FRN), is believed to be more sensitive to negative feedback, such as incorrectness or monetary losses (Falkenstein et al., 1991; Miltner et al., 1997; Gehring and Willoughby, 2002). The FRN, which peaks at around 250 ms after feedback onset, is maximal at frontal-central scalp electrode sites and is most likely generated in the anterior cingulate cortex (ACC) (Miltner et al., 1997; Gehring and Willoughby, 2002). The FRN is believed to reflect the binary evaluation of good vs. bad outcomes (Holroyd and Coles, 2002; Hajcak et al., 2006). The reverse inference that more negative FRN implies the encoding of more negative values has been used in social decision making studies, e.g., studies using unfair outcomes as feedback (Polezzi et al., 2008; Boksem and De Cremer, 2010).

The reinforcement learning (RL) theory of the FRN proposes that the FRN amplitude depends on the relation between actual and expected outcomes. Differences between actual and expected outcomes or prediction errors provide teaching signals to select advantageous actions (Sutton and Barto, 1998; Nieuwenhuis et al., 2004). Holroyd and Coles (2002) have proposed that the FRN was produced by the impact of reinforcement learning signals carried by the mesencephlic dopamine system in the ACC. In particular, this research indicated that when events are better than expected, the basal ganglia induce a phasic increase, whereas when events are worse than expected, the basal ganglia induce a phasic decrease. Therefore, differences in expectations of rewards should modulate the size of prediction error signals (Holroyd and Coles, 2002). However, regarding whether the magnitude of the FRN is also modulated by reward expectation, previous studies have demonstrated inconsistent findings.

In one study, researchers manipulated feedback frequency in a gambling task and found that the magnitude of FRN was larger for infrequent feedback than for frequent feedback (Holroyd et al., 2003). In this study, participants were informed about the frequency explicitly. However, Hajcak et al. (2005) found that the FRN did not differ as a function of expectancy in two experiments in which reward frequency information was either explicitly presented to subjects on a trial-by-trial basis or it is learned by participants in a block, suggesting that the FRN was insensitive to reward probability. In another study, Hajcak et al. (2007) found that the magnitude of the FRN was sensitive to violations of reward prediction, but this effect might depend on the close coupling of prediction and outcome: the FRN was sensitive to frequency only when participants made reward predictions after but not before gambling choices (Hajcak et al., 2007). This study suggests that the FRN was modulated by expectation only when the prediction was made following a response and immediately before receiving feedback. However, these studies mainly examined the FRN in the appetitive domain (involving only wins and no wins). It is currently unclear how the FRN responds to prediction errors in the aversive domain. Recently, an electroencephalography (EEG) study found that the probability of reward modulated neural responses to wins, but not to losses (Cohen et al., 2007). In addition, power and phase coherence values following wins but not losses were modulated by reward probability. These findings suggest that there may be different neural mechanisms underlying feedback processing in wins and in losses (Cohen et al., 2007).

According to the reinforcement learning theory, the FRN is associated with events that are worse than expected. However, this idea has been challenged in several previous studies. In one study, participants were asked to evaluate the correctness of their own response and were given the real feedback on their performance. The FRN was observed for both unexpected “correct” and “incorrect” feedbacks, regardless of the motivational value (Oliveira et al., 2007). It has been shown that midbrain dopamine neurons not only respond to rewards but also to transmit signals related to salient but non-reward experiences (Bromberg-Martin et al., 2010). Another study found that the FRN not only reflects a negative reward prediction error, but also a positive reward prediction error (Pfabigan et al., 2011). The researchers proposed that the FRN reflects the mismatch between internal and external representations and detects the motivational salience of outcomes (Pfabigan et al., 2011). A recent study found that unexpected physical pain omission (a positive reward prediction error) also yielded a more negative FRN than unexpected pain delivery, suggesting that FRN expresses salience prediction errors rather than reward prediction errors (Talmi et al., 2013). In addition, an EEG-functional magnetic resonance imaging (fMRI) study demonstrated that additional activations of the salience network and surprise signals modulate the FRN amplitude (Hauser et al., 2014), consistent with the recent theory that dopamine neurons also encode surprise-like saliency signals (Redgrave and Gurney, 2006; Matsumoto and Hikosaka, 2009; Bromberg-Martin et al., 2010).

Taken together, it is possible that the FRN encodes prediction errors associated with motivational salience rather than motivational value. The reason why FRN is typically more negative for losses than rewards probably might be that losses are more motivationally salient (Oliveira et al., 2007). In the present study, we designed conditioning learning experiments, involving both appetitive and aversive conditions, to address these underlying issues. We hypothesized that in appetitive condition, the FRN would be more negative in response to no win outcome (negative omitted outcome) than to win outcome (positive delivered outcome). But the FRN would be reversed in aversive conditions, with no losses (positive omitted outcome) outcome being more negative than loss outcome (negative delivered outcome). Such FRN patterns will present an important challenge for the RL theory. In the appetitive condition, the outcome was either win or no win. In the aversive condition, the outcome was either loss or no loss. The no win and no loss outcomes were “omitted” feedback, i.e., less than expected. We also sought to examine how explicit prediction modulates the sensitivity of the FRN to expectation of outcome.

## Materials and methods

### Participants

Fifteen healthy, right-handed participants (4 male; mean age ± SD, 20.93 ± 1.53 years) participated in return for payment. All participants had normal or corrected-to-normal vision, reported that they had no neurological or psychiatric disorders. All participants were right-handed, according to the Edinburgh Handedness Inventory (Oldfield, 1971). The study was approved by the Academic Committee of the School of Psychology at South China Normal University. All participants gave written, informed consent and were informed of their right to discontinue participation at any time.

### Experimental paradigm

**Figure 1:**
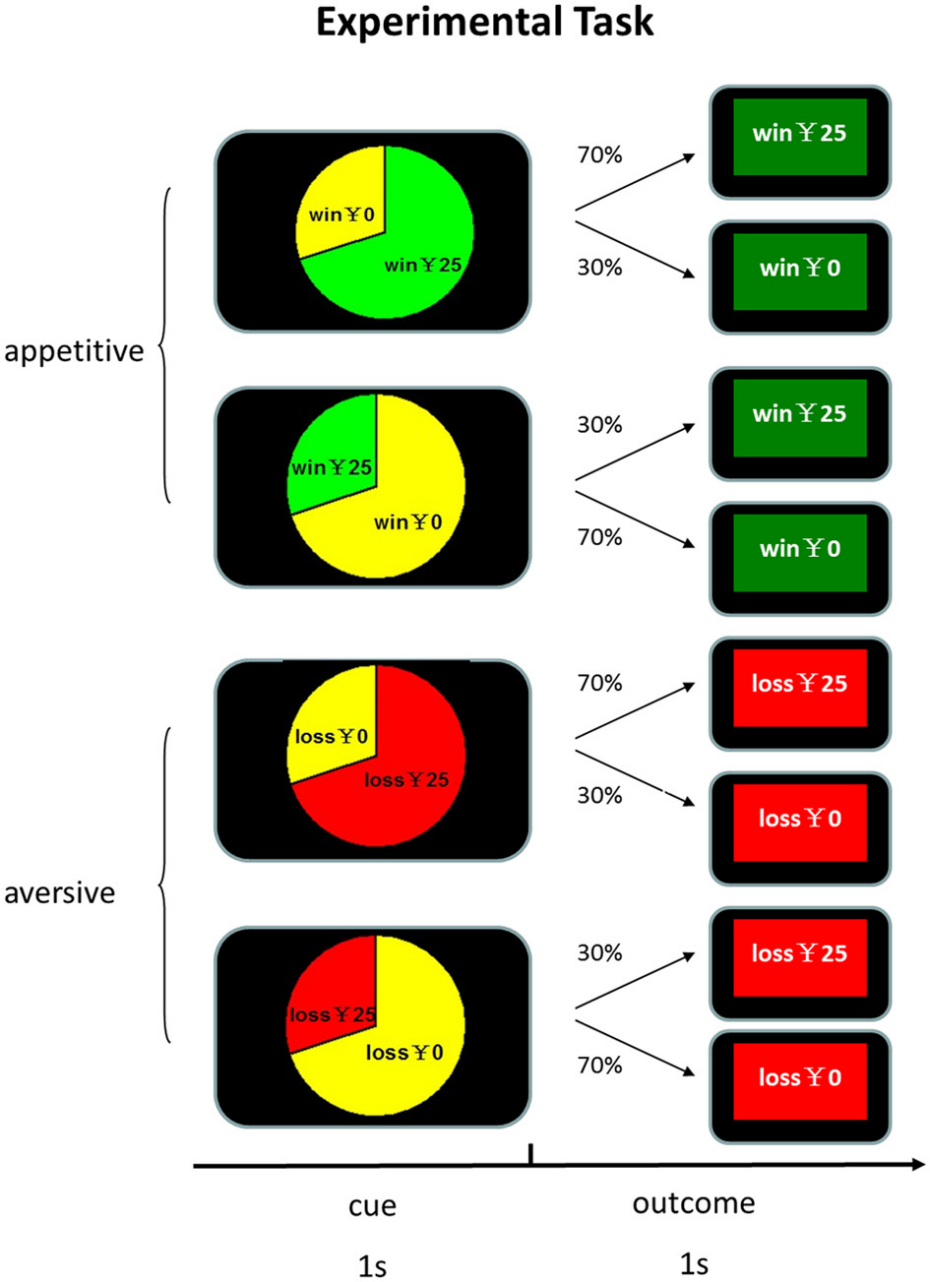
**Experimental task design**. Participants were presented with a pie chart which explicitly indicated the true frequencies of outcomes for 1 s. Then the feedback of outcome was shown for 1 s, followed by a 500 ms blank.

At the beginning of each trial, participants were presented with a pie chart which explicitly indicated the true frequencies of outcomes for 1 s. Then the feedback of outcome was shown for 1 s, followed by a 500 ms blank. There were 4 kinds of outcomes: win, no win, lose and no loss. We varied the amount of reward in order to entertain subjects so that they can maintain a high level of vigilance and interest in the task. The amount of winning and losing varied from ¥10 to ¥39, with an increment of ¥1. The win and no win feedbacks in the appetitive condition were highlighted with green background and the loss and no loss feedbacks in the aversive condition were highlighted with red background. The assignment of background color to experimental conditions was counterbalanced across participants. In order to increase participants' engagement, they were required to confirm the type of feedbacks by pressing the corresponding keys within 1 s. Unknown to the participants, the association between cues and feedback was pre-determined and cue identity and experimental conditions were counterbalanced across participants. As shown in Figure 1, in the appetitive condition in which only wins and no win were possible, one cue was associated with reward with 70% probability and the other cue was associated with reward with 30% probability. In the aversive condition in which only losses and no loss were possible, one cue was associated with losses with 70% probability and the other was associated with losses with 30% probability. The experiment was consisted of three blocks of 200 trials each. The two conditions were randomized across blocks and participants. There were 100 “appetitive condition” trials and 100 “aversive condition” trials in each block. All the participants received a base payment of 30 yuan (about 5 US dollar).

After the EEG session, participants were required to indicate their feelings of surprise about the eight types of outcomes on a 10-point Likert scale. After completion of the experiment, one trial was randomly selected and the outcome was multiplied by a rate (0.5) which was unknown to subjects before the experiment. The amount of winning was added to the base payment. The amount of losing was subtracted from the base payment.

### ERP recording and analysis

Standard ERP recording and analysis were applied. EEGs were recorded from 32 scalp sites using Ag/AgCl electrodes embedded in an elastic cap (NeuroScan Inc., USA) according to the international 10–20 system, with the reference to the right mastoid. Eye blinks were recorded from electrodes located above and below the left eye. The horizontal electro-oculogram (EOG) was recorded from electrodes placed 1.5 cm lateral to the left and right external canthi. All electrode impedances were maintained below 5 kΩ. The EEG and EOG were recorded from SynAmps AC amplifiers using a 0.5–70 Hz bandpass and continuously sampled at 500 Hz/channel for off-line analysis.

The EEG data were re-referenced offline to linked-mastoid electrodes by subtracting 50% of the signal in the right mastoid electrode from the signal in each channel. Ocular artifacts were corrected with an eye-movement correction algorithm (Gratton et al., 1983). All trials in which EEG voltages exceeded a threshold of ±70 μV during the recoding epoch were excluded from analysis. The EEG data were filtered using a 1–20 Hz band-pass (24 dB octave roll off) for the FRN. The EEG data were baseline corrected by subtracting from each sample the average activity of that channel during the baseline period. Epochs of 800 ms (with 200 ms pre-stimulus baseline) EEG from each electrode were time-locked to the onset of feedback and were sorted by experimental conditions.

The FRN was measured as the mean amplitude in the time window 200–320 ms post-onset of the outcome stage. We focused on the FRN responses on the anterior frontal midline electrodes (Fz), since the FRN effects were the largest on this electrode. Less than 5% of epochs were excluded in each condition in three experiments.

## Results

### Behavioral results

**Figure 2:**
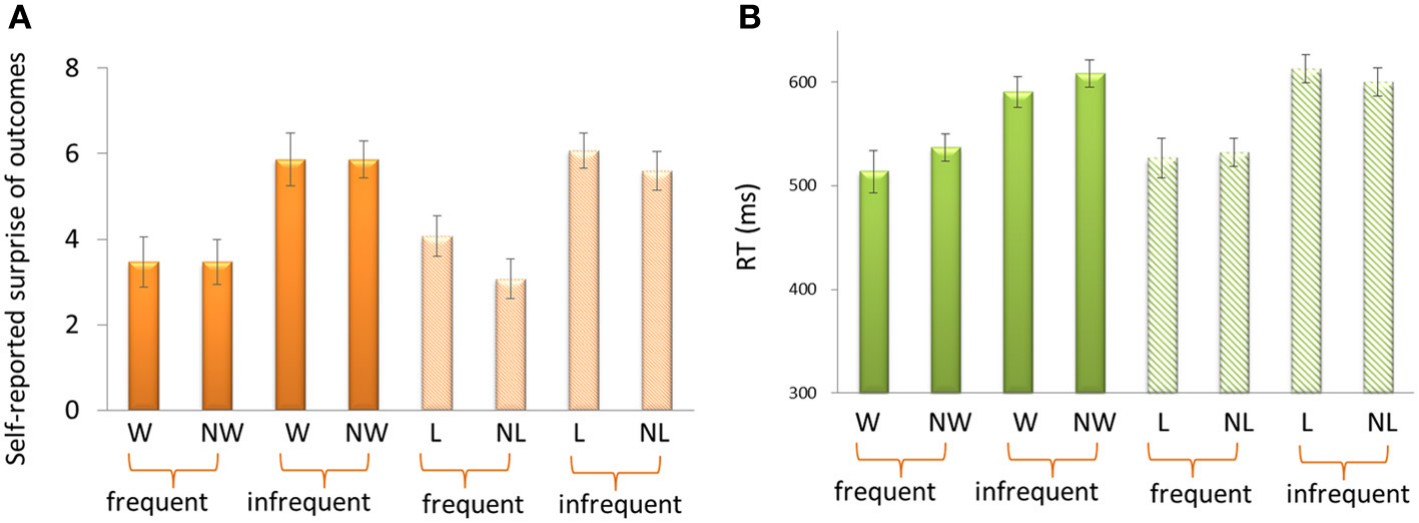
**Self-reported surprise (A) and reaction times (B) in response to outcomes**. W, win; NW, no win; L, loss; NL, no loss.

For the self-reported surprise in response to outcomes (Figure 2A), the 3-Way repeated-measure ANOVA using frequency (70 vs. 30%), outcome (“omitted” feedback vs. “delivered” feedback) and domain (appetitive vs. aversive) as independent factors revealed a significant main effect of frequency, *F*_(1, 14)_ = 22.33, *p* < 0.001. Participants felt more surprise for infrequent outcomes (5.85 ± 0.31) than for frequent outcomes (3.52 ± 0.0.53). The other effects were not significant, *p* > 0.1.

For the reaction times (RTs) (Figure 2B), the 3-Way ANOVA revealed a significant main effect of frequency, *F*_(1, 14)_ = 33.75, *p* < 0.001. Participants reacted more quickly for frequent outcomes (527.50 ms ± 15.46) than for infrequent outcomes (602.94 ms ± 11.98). We also found a significant interaction effect between domain and outcome, *F*_(1, 14)_ = 5.56. *p* = 0.034. The difference between delivered outcomes and omitted outcomes was larger in appetitive domain (−20.49 ± 10.80) than in aversive domain (3.44 ± 9.80). The other effects were not significant, *p* > 0.1.

### ERP results

**Figure 3:**
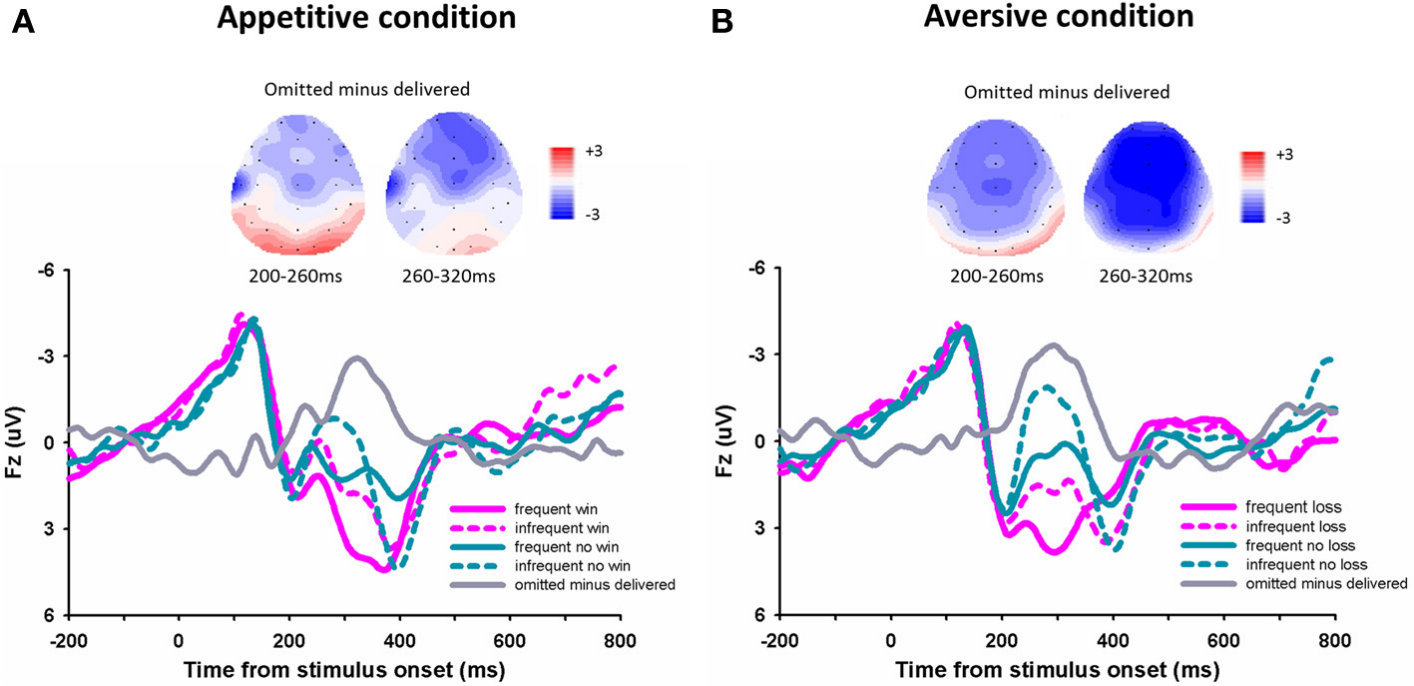
**The ERP grand-average waveforms and topographical maps**. Grand-average waveforms and different waveforms at channel Fz for the appetitive condition **(A)** and the aversive condition **(B)**.

For the outcome evoked FRN (Figures 3A,B), the 3-Way repeated- measures ANOVA using frequency (70 vs. 30%), outcome (“omitted” feedback vs. “delivered” feedback) and domain (appetitive vs. aversive) as independent factors revealed a significant main effect of outcome, *F*_(1, 14)_ = 31.94, *p* < 0.001, with more negative FRN responding to omitted feedbacks (0.32 μV ± 0.44) than to delivered feedbacks (2.07 μV ± 0.45). The main effect of frequency was also significant, *F*_(1, 14)_ = 16.71, *p* = 0.001, with more negative FRN responding to infrequent outcomes (0.57 μV ± 0.46) than to frequent outcomes (1.82 μV ± 0.42), suggesting that the FRN was sensitive to the expectation of outcomes. The main effect of domain was also significant, *F*_(1, 14)_ = 5.65, *p* = 0.032, with more negative FRN responding to the appetitive domain than to the aversive domain. The interaction effect between domain and outcome was significant, *F*_(1, 14)_ = 12.80, *p* = 0.003. The difference of FRN amplitude between omitted feedbacks and delivered feedbacks was larger in aversive condition (−2.42 μV ± 0.32) than in appetitive condition (−1.08 μV ± 0.40). The other effects were not significant, *p* > 0.05.

## Discussion

In the present study, we examined whether and how the FRN evaluates the valence (delivered feedback and omitted feedback) and expectation of feedback in appetitive and aversive conditions. We found that the FRN was sensitive to the valence of feedback in both appetitive and aversive conditions. Contrary to the finding of (Holroyd et al., 2004), the FRN we obtained is more negative for omitted outcomes than delivered outcomes in both appetitive and aversive conditions. In appetitive context, the FRN was more negative in the no win condition (i.e., “omitted” feedback) than in the win condition. Importantly, in aversive context, the FRN was more negative in the no loss condition (i.e., “omitted” feedback) than in the loss condition. We also found that the FRN was strongly modulated by expectation when the frequencies of outcomes were explicitly indicated by cues. The FRN elicited by infrequent conditions were more negative than frequent conditions in both appetitive and aversive contexts.

The FRN has been used as an index of negative feedback processing since the majority of studies showed more negative FRN for the worse available outcomes. However, our study demonstrated that the no loss feedback elicited more negative FRN than loss, although no loss is clearly better than loss. Our findings are in line with a recent study which found that the FRN expresses salience prediction errors rather than reward prediction errors (Talmi et al., 2013). In that study, the appetitive (i.e., monetary reward) and aversive (i.e., physical pain) conditions were presented in different blocks. In the reward session, they found that unexpected reward omissions (a negative reward prediction error) yielded a more negative deflection relative to unexpected reward delivery. In the pain session, they found that unexpected pain omission (a positive reward prediction error) also yielded a negative deflection relative to unexpected pain delivery. Monetary gain in appetitive condition is a secondary reinforcer, while pain in the aversive condition is a primary reinforcer. Thus, difference in reward and pain may be due to the difference in primary and secondary reinforce rather than to the difference in appetitive/aversive domain. One advantage of our design is that we used money as the only modality which makes the appetitive and aversive conditions more comparable. Moreover, we presented gains/losses in the same block rather than separate them into different sessions. Our study strengthens the notion that the FRN encodes salience prediction error (“more or less”) rather than reward prediction error (“better or worse”).

Our current study, together with several previous studies, suggests that caution in the interpretation of the FRN as an index of negative values should be applied. The RL theory proposed that the FRN reflected the impact on ACC of a negative reward prediction error signal, conveyed by the midbrain dopamine system that was generated when ongoing events were worse than expected (Holroyd and Coles, 2002). However, in previous two studies, the FRN amplitudes elicited by negative and neutral outcomes did not differ, but the FRN associated with “omitted” feedback was larger than the negative feedback (Hajcak et al., 2006; Holroyd et al., 2006). Moreover, another research found that in the loss condition, the FRN associated with loss 0 was more negative than loss 5 and the difference was significant (Yu and Zhou, 2006). A previous fMRI study also showed that, striatal activation reflected positively signed prediction errors for both appetitive and aversive, suggesting that appetitive and aversive prediction errors may be represented in a similar manner, albeit somewhat spatially resolvable along an axis of the striatum (Seymour et al., 2007). Our findings are consistent with the findings that the neurons in macaque dorsal ACC were more activated in response to both large and small rewards when the outcomes were surprising. These results are inconsistent with the notion that the ACC encodes error signals in reward prediction (Hayden et al., 2011). These patterns are similar to that predicted in attentional theories of learning whereby the rate of learning is modulated by the amount of attention elicited by the outcomes. The associations between actions and subsequent rewards are stronger when outcomes are surprising, regardless of their valence. (Pearce and Hall, 1980; Roesch et al., 2010).

A computational model of salience suggests that a signal for certain rewards is more salience than a signal for certain non-reward (Esber and Haselgrove, 2011). This implies that delivered outcome is more salient than omitted outcome. In appetitive condition, the prediction of participants was “win,” the “omitted” feedback was less than expected (i.e., absence in expected). Therefore the FRN elicited by “omitted” feedback was more negative than elicited by win feedback. Similarly, in aversive condition, the prediction of participants was “loss,” the “omitted” feedback was less than expected. Thus, the FRN elicited by “omitted” feedback was more negative than elicited by loss feedback. No matter in appetitive condition or aversive condition, the “omitted” feedback was a neutral outcome indicating that the salient wins or losses were absent. It is possible that the FRN reflects “less than expected” prediction error signal rather than “worse than expected” signal. We propose that the ACC evaluative system that produces the FRN might classify outcomes in a “more-or-less than expected” fashion rather than in the “better-or-worse than expected” dimension.

A few caveats about the present study should be mentioned. First, our study did not capture the dynamics of participants' prediction. The precise prediction may vary from trial to trial. Future studies may use computational modeling to estimate the exact prediction on a given trial. However, in the present study, we only associated average ERP amplitude with average prediction in a given trial type. We assume that participants will expect win in high probability win trials, although the strength of such expectation may differ across trials. Future studies may use computational modeling combined with single trial ERP analysis to further investigate the neural correlates of such dynamic predictions (Chase et al., 2011; Walsh and Anderson, 2011). Second, in our task, subjects were instructed to press a button during the outcome period and the frontocentral ERP may be associated with planning and preparation of the motor action. However, our topographic maps of the FRN suggest that the FRN was mainly generated in frontocentral regions, e.g., the ACC. Future studies may use a passive observation task to rule out the confounding effects of motor planning and actions. Finally, our study did not examine the correlation between the prediction error and FRN on a trial-by-trial basis. Previous studies have shown that the FRN amplitude was correlated positively with negative prediction error which was derived from a reinforcement learning model (Chase et al., 2011). Our study only adopted two levels of probability (30 and 70%). Future studies may vary reward probability across its full range and use single trial analyses to test whether the FRN scales with increasing magnitude of prediction error (Pernet et al., 2011).

In conclusion, our study provide evidence that the FRN is not necessarily associated with negative feedback and may reflect the “more or less than expected” signal, e.g., delivered or omitted. These findings, if replicated, challenge the generally accepted notion that the FRN encodes negative feedback and “worse than expected” prediction error and raise awareness as to the need to revise our current understanding of the functional significance of the FRN.

### Conflict of interest statement

The authors declare that the research was conducted in the absence of any commercial or financial relationships that could be construed as a potential conflict of interest.
